# Concurrent insulinoma with mosaic Turner syndrome: A case report

**DOI:** 10.3892/etm.2015.2167

**Published:** 2015-01-05

**Authors:** SHAOYUN WANG, LIJUAN YANG, JIE LI, YIMING MU

**Affiliations:** 1 Department of Endocrinology and Metabolism, Chinese PLA General Hospital, Chinese PLA Medical College, Beijing 100853, P.R. China; 2 Department of Pathology, Chinese PLA General Hospital, Chinese PLA Medical College, Beijing 100853, P.R. China

**Keywords:** Turner syndrome, mosaic 45XO/47XXX, insulinoma, premature ovary failure

## Abstract

Turner syndrome is a chromosomal abnormality in which the majority of patients have a 45XO karyotype, while a small number have a 45XO/47XXX karyotype. Congenital adrenal hyperplasia has been previously reported in patients with Turner syndrome. Although insulinomas are the most common type of functioning pancreatic neuroendocrine tumor and have been reported in patients with multiple endocrine neoplasias, the tumors have not been reported in patients with mosaic Turner syndrome. The present study reports the first case of an insulinoma in a patient with 45XO/47XXX mosaic Turner syndrome. The patient suffered from recurrent hypoglycemia, which was relieved following ingestion of glucose or food. A 5-h glucose tolerance test was performed and the levels of glucose, C-Peptide and insulin were detected. In addition, computed tomography (CT) and ultrasound scanning were performed to evaluate the possibility of an insulinoma. Pathological examination and karyotyping were performed on a surgical specimen and a whole blood sample, respectively. The patient was found to suffer from premature ovarian failure, and a physical examination was consistent with a diagnosis of Turner syndrome. An ultrasound scan demonstrated streak ovaries and the patient was found to have a 45XO/47XXX karyotype. Furthermore, a lesion was detected in the pancreas following CT scanning, which was identified as an insulinoma following surgical removal and histological examination. In conclusion, the present study reports the first case of an insulinoma in a patient with mosaic Turner syndrome. Since mosaic Turner syndrome and insulinoma are rare diseases, an association may exist that has not been previously identified.

## Introduction

Turner syndrome is a chromosomal abnormality, affecting one in 2,000 female live-births (1). X-chromosome monosomy (45XO karyotype) accounts for >55% of Turner syndrome cases. Mosaicism, represented mainly by 45XO/46XX or 45XO/47XXX, has been detected in the remaining cases, with only 1–4% of the reported cases being 45XO/47XXX (2,3).

Patients with Turner syndrome present with a number of symptoms, including short stature, gonadal failure, broad chest, low hair-line, low-set ears and a webbed neck. Medical problems associated with Turner syndrome include congenital heart disease, hypothyroidism, diabetes, vision and hearing problems, cognitive deficits and autoimmune diseases. Mosaics with a normal cell line or an extra X chromosome tend to exhibit a milder phenotype. In addition, patients with 45XO/47XXX may not develop mental or behavioral problems and have a higher fertility rate (4). A previous study reported a case of congenital adrenal hyperplasia associated with Turner syndrome (5).

Insulinomas are the most common type of functioning pancreatic neuroendocrine tumor (PNET), with an incidence of two to four cases per million individuals each year. Insulinomas usually result in recurrent episodes of fasting hypoglycemia and tend to be small with no mass effect (6). Although insulinomas are often sporadic, they account for 10–30% of functioning PNETs in patients with multiple endocrine neoplasia type 1. In addition, these tumors have been reported in patients with neurofibromatosis type 1 (7,8). However, insulinomas have not been previously reported in patients with mosaic 45XO/47XXX Turner syndrome or other forms of Turner syndrome. To the best of our knowledge, the present study reports the first case of a pathologically confirmed insulinoma in a patient with mosaic 45XO/47XXX Turner syndrome. Due to the rarity of the two diseases, the current case may represent a previously unrecognized association.

## Case report

The present case study was approved by the Ethics Committee of PLA General Hospital (Beijing, China), and informed consent was obtained from the patient. A 30-year-old female presented with ‘episodic dizziness, sweating and loss of consciousness’ in April 2012. The patient was admitted to the Chinese PLA General Hospital (Beijing, China) with deteriorating symptoms. The patient was found to suffer from fasting hyperinsulinemic hypoglycemia (blood glucose, 2.8 mmol/l), which improved following the ingestion food high in glucose. Within the 13-month period following the initial onset of symptoms, the patient gained ~5 kg.

Medical history review revealed an allergy to iodine contrast dye. The patient experienced menarche at the age of 13 years and had regular monthly menses until the age of 23 years. Despite treatment with progesterone and estrogen, the last spontaneous menstrual period of the patient was on the 10^th^ of December 2011. The patient had been sexually active from the age of 23 years, but did not conceive despite regular unprotected sexual activity. In addition, the female was a labor worker and did not smoke or drink alcohol. There was no family history of Turner syndrome or other genetic diseases, with the exception of a parent having short limbs.

Physical examination revealed a short stature (152 cm), a webbed neck, short limbs, an abnormal upper-to-lower segment ratio, a low posterior hair line with acanthosis nigricans behind the neck and a body mass index of 33.3 kg/m^2^. External examination of the patient’s genitalia revealed no abnormalities, a smooth vagina and a small uterus that was positioned forward, but was movable without tenderness. The female was found to have an IQ within an average range according to the Wechsler Adult Intelligence Scale (9).

**Figure 1:**
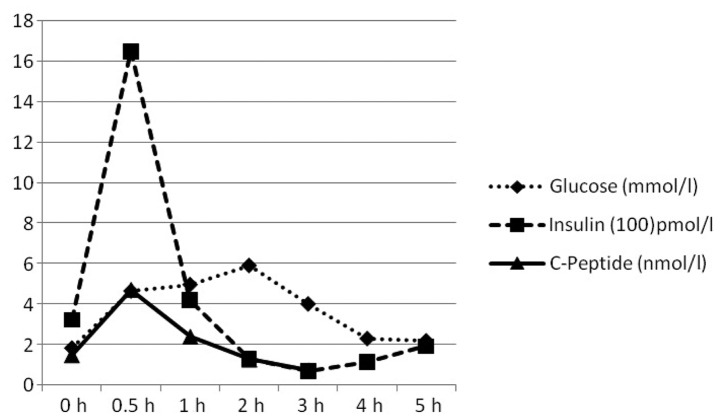
Glucose, insulin and C-peptide levels upon oral administration of 75 g glucose. The glucose concentration decreased when the patient was fasting and at time points after 2 h from the oral glucose administration. Normal ranges: Glucose, 4.4–6.1 mmol/l; Insulin, 17.80–173 pmol/l; C-peptide, 0.370–1.470 nmol/l.

The patient was subjected to an oral glucose tolerance test, where 75 g glucose was administered and blood samples were obtained at baseline, 0.5, 1, 2, 3, 4 and 5 h to detect the serum levels of glucose, insulin and C-peptide. Serum results revealed that the fasting serum glucose level was low, while the levels of insulin and C-peptide were high (Fig. 1). The inulin/glucose ratio was consistently >0.3, indicating the possibility of an insulinoma.

The estrogen level was found to be slightly decreased, whereas the follicle-stimulating hormone and luteinizing hormone levels were slightly elevated. In addition, the levels of thyroid hormone, thyroid-stimulating hormone and growth hormone in the patient were found to be normal. Furthermore, the patient’s cortisol level was slightly reduced at 8 am, while the adrenocorticotropic hormone level was slightly elevated. Antibodies for insulin or islet cells were not detected. A magnetic resonance imaging scan revealed a normal pituitary gland, and the chest X-ray scan appeared to be normal. An ultrasound scan demonstrated that the left ovary was 1.4×0.9×0.6 cm^3^, the right ovary was 1.3×0.5×1.0 cm^3^ and the two ovaries were streak-like, without visible follicles.

**Figure 2:**
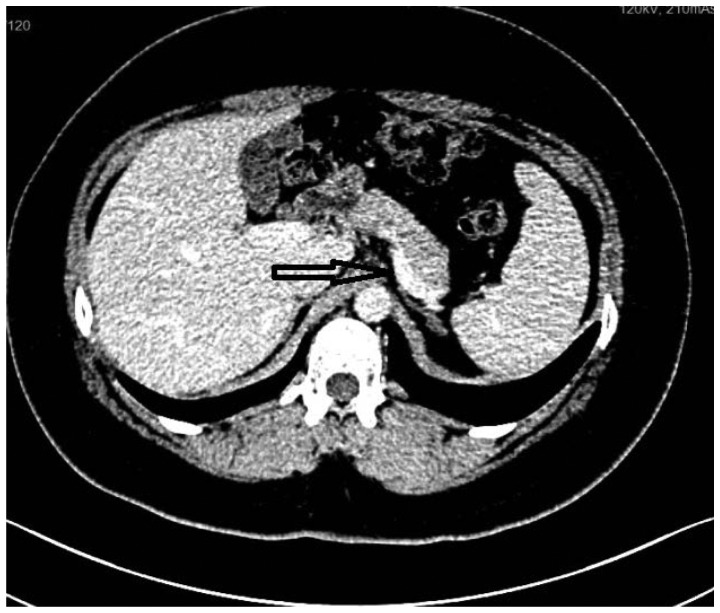
Computed tomography scan of the pancreas. An enhanced lesion was observed around the tail of the pancreas, which was well-defined, homogeneously distributed and had dimensions of ~2.1×1.1 cm, indicating a possible benign lesion.

**Figure 3:**
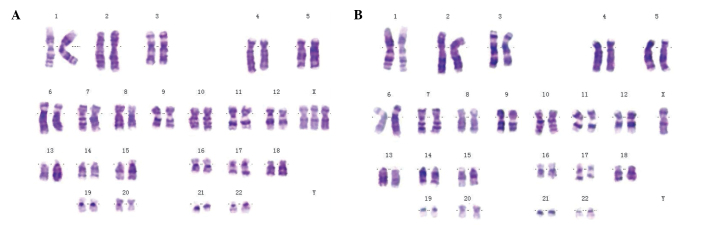
Karyotyping of a whole blood sample from a patient with a 45XO/47XXX karotype, with (A) 68% 45XO and (B) 32% 47XXX.

An endoscopic ultrasound revealed a low-echo, dense lesion at the junction of the pancreas body and tail, with dimensions of 2.0×1.28 cm^2^. The observations were confirmed with ultrasonic angiography and enhanced computed tomography scanning of the pancreas, indicating the possibility of an insulinoma (Fig. 2). The karyotype of the patient was found to be 45XO/47XXX from a whole blood sample, with 68% 45XO and 32% 47XXX (Fig. 3).

**Figure 4:**
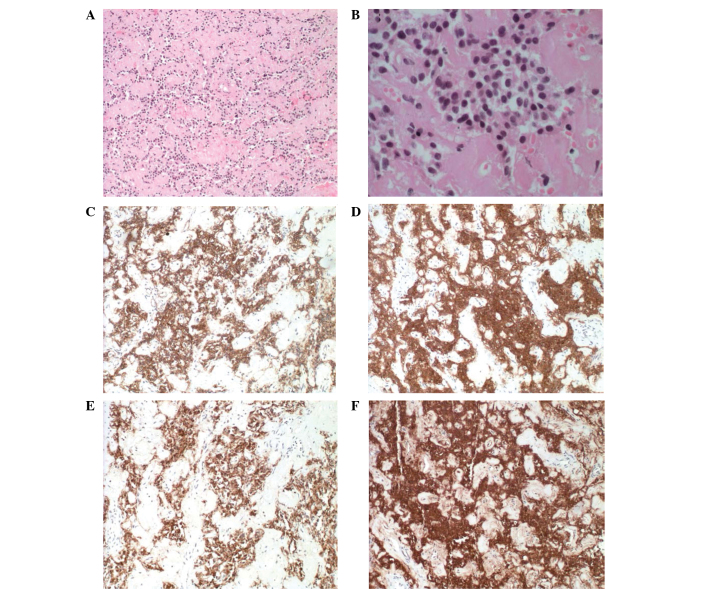
Pathological and immunohistochemical results of an insulinoma lesion removed from the patient. The lesion is presented with a magnification of (A, C, E) ×100 and (B, D, F) ×400. The tumor appeared to be positive for (C) β-catinin (++), (D) CD56 (++), (E) insulin (++) and (F) synaptophysin (+++).

Subsequently, the patient was transferred to the Department of Surgery at the Chinese PLA General Hospital and the lesion was removed under general anesthesia per standard protocol (10). Pathological examination revealed a grade 1 neuroendocrine lesion that was well-differentiated. Immunochemical staining revealed that the tumor was positive for synaptophysin (+++), CD56 (++), insulin (++), creatine kinase (+) and β-catinin (++), but negative for glucagon (−), CD10 (−) and Ki-67 (Fig. 4). Immediately following surgical removal of the insulinoma, the blood glucose level of the patient increased to 9.19 mmol/l, which was reduced to 6.0 mmol/l within four days of surgery. The patient’s symptoms were markedly resolved following surgery.

## Discussion

To the best of our knowledge, the present study is the first case report of an insulinoma in an individual with 45XO/47XXX mosaic Turner syndrome.

An increased number of X chromosomes has been shown to reduce the extent of ovarian failure and create a period of potential fertility (4,11–13). The patient in the present case study experienced normal menses for ~10 years, between the age of 13 and 23 years. For patients with mosaic Turner syndrome, during the time in which they have normal menstruation, they may be fertile (14–16).

Turner syndrome is commonly associated with diabetes and insulin resistance. Although a previous study supports the genetic basis for the declined β-cell function, the exact mechanism of the disease remains unclear (17). Hypoglycemia in Turner syndrome is rare. A previous case study reported the case of an infant with Turner syndrome who suffered from hypoglycemia due to a growth hormone deficiency (18,19). By contrast, the patient in the present study showed a normal growth hormone level.

Insulinomas and Turner syndrome are relatively rare conditions. The occurrence of two rare conditions simultaneously may be due to coincidence. However, since insulinomas and mosaic Turner syndrome are caused by changes to the same allele on chromosome 11, a true association may exist that has not been previously recognized. In the case that a patient presents with fasting hypoglycemia, the possibility of an insulinoma should be always considered. However, the convergence of the two rare conditions should sensitize the consideration that in the context of mosiac Turner syndrome, an insulinoma may be more common.
